# Microbial cell factory for butyl butyrate production: Knowledges and perspectives

**DOI:** 10.1111/1751-7915.14502

**Published:** 2024-06-18

**Authors:** Xiaolong Guo, Yuqing Ding, Yufan Chen, Hongxin Fu, Jufang Wang

**Affiliations:** ^1^ School of Biology and Biological Engineering South China University of Technology Guangzhou China; ^2^ Guangdong Provincial Key Laboratory of Fermentation and Enzyme Engineering South China University of Technology Guangzhou China; ^3^ State Key Laboratory of Pulp and Paper Engineering South China University of Technology Guangzhou China

## Abstract

Butyl butyrate is a short‐chain fatty acid ester (C8) with a fruity aroma. It has broad prospects in the fields of foods, cosmetics and biofuels. At present, butyl butyrate is produced by chemical synthesis in the industry, but it is highly dependent on petroleum‐based products. The growing concerns regarding the future scarcity of fossil fuels have been strongly promoted the transition from traditional fossil fuels and products to renewable bioenergy and biochemicals. Therefore, it is necessary to develop a green biochemical technology to replace traditional petroleum‐based materials. In recent years, microorganisms such as *Escherichia coli* and *Clostridium* have been engineered to serve as cell factories for the sustainable one‐pot production of short‐chain fatty acid esters, including butyl butyrate. This opinion highlights the recent development in the use of lipases and alcohol acyltransferases (AATs) for butyl butyrate production in microbial fermentation, as well as future perspectives.

## Graphical abstract

Butyl butyrate is a C8 short‐chain fatty acid ester with a fruity aroma, which has broad prospects in the food and chemical industries. This opinion highlights the recent development in the use of lipases and alcohol acyltransferases (AATs) for butyl butyrate production in microbial fermentation, as well as future perspectives.

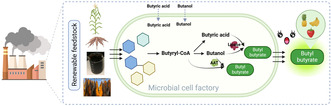

## Introduction

Butyl butyrate, one of the short‐chain fatty acid esters, has broad prospects in the food and chemical industries, as well as in renewable energy as a jet fuel or as an additive for gasoline and diesel to improve their octane rating and combustion efficiency (Guo et al., 2023). The demand for esters has been increasing in recent years with the continuous expansion of its application range. The global market demand for esters, including butyl butyrate, is predicted to reach $ 159.36 billion by 2033, with a compound annual growth rate (CAGR) of 5.4% (Future Market Insights, 2023).

Butyl butyrate is naturally produced by plants, such as flowers and fruits (Xin et al., 2019). However, the concentration is relatively low when extracted from plants. Thus, to meet the demand, the industrial production of butyl butyrate is carried out by chemical synthesis, including Fischer esterification process with butyric acid and butanol as substrates. This method has been abandoned due to the awful reaction condition (requires high temperature–200°C and concentrated sulfuric acid), environmental pollution and equipment corrosion (Zhang et al., 2017). To overcome such issues in butyl butyrate production, mild reactions, such as Tishchenko reaction and Acetylation are employed at room temperature (Kushwaha et al., 2022). Nevertheless, these reactions still rely on the petroleum refining industry. With the increasing depletion of petrochemical resources, the increasing environmental pollution caused by traditional energy consumption and the public's preference for bio‐based chemicals, there is an urgent need to develop biosynthesis of butyl butyrate.

Biosynthesis of butyl butyrate mainly includes lipases and alcohol acyltransferases (AATs) dependent pathways (Noh et al., 2019). If these pathways are introduced into microorganisms, they can produce butyl butyrate from glucose or other sugars in one pot. At present, microorganisms such as *Escherichia coli* (Layton and Trinh, 2014; Lee and Trinh, 2022) and *Clostridium* (Feng et al., 2021; Guo et al., 2023; Noh et al., 2018) have been engineered to serve as cell factories for butyl butyrate production.

In this opinion, the strategies for butyl butyrate production via lipases and AATs pathways are reviewed. In addition, the future perspectives and research directions for high concentration, yield, productivity and low cost of renewable butyl butyrate production are suggested.

## LIPASE‐DEPENDENT BUTYL BUTYRATE PRODUCTION

Lipase (EC 3.1.1.3) is an esterase that catalyse the conversion of organic acids and alcohols into esters following a ping‐pong bi‐bi mechanism (Chulalaksananukul et al., 1990). Currently, the lipase was harnessed for butyl butyrate production in microbial fermentation. Some natural strains are capable of producing butyl butyrate without the addition of any precursors and lipases. For example, *Clostridium* sp. strain BOH3 (Xin et al., 2016) and *Clostridium acetobutylicum* NJ4 (Lu et al., 2023), which naturally produced butyric acid and butanol as the precursors of butyl butyrate and a lipase as the catalyst for butyl butyrate synthesis, could produce 6 and 1 g/L butyl butyrate, respectively. To elevate butyl butyrate production, the precursor butyric acid was added exogenously to the *C. acetobutylicum* NJ4 fermentation broth, resulting in 18 g/L butyl butyrate production (Lu et al., 2023). Furthermore, the precursor butyric acid/butanol was supplemented exogenously into the fermentation broth along with lipase for further improvement of butyl butyrate production. By supplementing lipase and butyric acid, the butanol producing strain *Clostridium* sp. strain BOH3 (Xin et al., 2016) produced 22 g/L butyl butyrate. By supplementing lipase and butanol, the butyric acid‐producing strain *Clostridium tyrobutyricum* ATCC 25755 produced 35 g/L butyl butyrate (Zhang et al., 2017), which was the highest titre of butyl butyrate production achieved using lipase as the catalyst. To avoid adding exogenous precursors butyric acid and butanol, the co‐culture system, containing a butanol‐producing strain and a butyric acid‐producing strain such as *Clostridium beijerinckii* BGS1–*C. tyrobutyricum* ATCC 25755 (Cui et al., 2020), *C. acetobutylicum* NJ4–*C. tyrobutyricum* LD (Lu et al., 2023), and *E. coli*–*E. coli* (Sinumvayo et al., 2021), produced 5–34 g/L butyl butyrate.

## AAT‐DEPENDENT BUTYL BUTYRATE PRODUCTION

Alcohol acyltransferases (AATs), primarily found in yeast, fruits and flowers, are a class of enzymes that catalyse the condensation of acyl‐CoA and alcohol to form esters (Guenther et al., 2011; Mason & Dufour, 2000; Shalit et al., 2003). The AATs commonly used in the production of butyl butyrate are SAAT, VAAT and AAAT (Feng et al., 2021; Noh et al., 2018). In *E. coli*, by introducing butyryl‐CoA and butanol synthetic modules and a SAAT module, the engineered strains produced 21–450 mg/L butyl butyrate (Layton & Trinh, 2014, 2016a, 2016b; Lee & Trinh, 2022). An *E. coli*–*C. beijerinckii* G117 co‐culture system, in which *E. coli* (overexpressing *ctfAB* and *VAAT*) converted butyric acid and butanol produced by *C. beijerinckii* G117 into butyl butyrate, generated 1280 mg/L butyl butyrate (Cui et al., 2023).

To improve butyl butyrate production, the anaerobic *Clostridium*, harbouring native pathways for precursors butyryl‐CoA and butanol synthesis, was used as the host. In 2018, Noh et al., attempted to produce butyl butyrate with a de novo synthetic approach by overexpressing AATs in *C. acetobutylicum*. The engineered strains CaSAAT and CaAAAT produced 40–50 mg/L butyl butyrate (Noh et al., 2018). After that, *Clostridium saccharoperbutylacetonicum* N1‐4‐C was engineered by eliminating prophages and overexpressing of *sadh‐hydG* and *SAAT*. As a result, the engineered strain produced 2 g/L butyl butyrate (Feng et al., 2021). Additionally, *C. tyrobutyricum* has been found to produce the highest titre of butyl butyrate when using lipase as the catalyst. It was later confirmed as a dark horse for butyl butyrate production. For example, by introducing the butanol and butyl butyrate synthetic pathways, regulating the expression level of key genes, increasing the concentration of the precursors (butyryl‐CoA and butanol), optimizing the fermentation temperature, and adopting mannitol as a substrate, the engineered strain produced 63 g/L of butyl butyrate from mannitol without the addition of any precursors and lipases (Guo et al., 2023). By further reinforcing the butanol synthetic pathway, weakening the butyric acid synthetic pathway, and optimizing the fermentation conditions (organic nitrogen concentration and two‐stage temperature shift), the final strain produced 143 g/L butyl butyrate (Guo, Ye, et al., 2024), which was the highest butyl butyrate production reported so far.

## CONCLUSIONS AND PERSPECTIVES

Although the use of lipase as the catalyst could produce a high concentration of butyl butyrate, this reaction has a relatively low equilibrium constant in the aqueous phase at room temperature, and is not thermodynamically favourable with a positive Gibbs free energy change (∆G), resulting in high substrate concentrations required to drive the reaction forward. Conversely, AAT catalytic reaction has a high equilibrium constant and is thermodynamically favourable which means relatively low substrate concentrations are needed to push the reaction forward (Seo et al., 2020). From an economic perspective, the addition of precursors and/or lipase not only increases the cost of substrates and enzymes but also raises downstream separation cost. Therefore, to achieve economical production of butyl butyrate, a de novo synthetic approach should be adopted. The natural butyl butyrate synthetic strain *Clostridium* sp. strain BOH3 was able to produce 6 g/L target product without the addition of any precursors and lipases (Xin et al., 2016). However, when the AAT pathway was adopted, the butyl butyrate produced by *C. tyrobutyricum* engineered strain was 23 times higher than that of *Clostridium* sp. strain BOH3 (Guo, Ye, et al., 2024). Therefore, when compared to lipase, the AAT‐dependent approach is more promising and attractive for butyl butyrate biosynthesis in microorganisms.

Although *E. coli* is a commonly used strain for natural compounds production, its ability to produce butyl butyrate is much lower than that of *Clostridium*. Butyryl‐CoA and its derivatives, butyric acid and butanol, are mainly produced by clostridia anaerobically, such as *C. tyrobutyricum*, which can produce more than 50 g/L butyric acid (Guo et al., 2020) or 25 g/L butanol (Zhang et al., 2018). Thus, anaerobic *Clostridium* is the preferred host for butyl butyrate production. *C. tyrobutyricum* is the most promising *Clostridium* strain for industrial bio‐based butyl butyrate production, with a yield of 0.28 mol/mol in fermentation. This is nine times higher than that of *C. saccharoperbutylacetonicum* (Feng et al., 2021; Guo, Ye, et al., 2024) but still only 56% of the maximum theoretical yield, which is still not comparable to the chemical synthesis. Future work should focus on eliminating by‐products, engineering cofactor and energy, regulating key genes, and optimizing fermentation conditions. On the other hand, almost all of the metabolic engineering strategies were based on previous studies due to the lack of understanding of the metabolic mechanism and metabolic regulatory network or pathway. Therefore, future studies should incorporate transcriptomics, metabolomics and/or proteomics to analyse the key elements, enzymes, and intermediate metabolites in the metabolic pathway. Meanwhile, the genome‐scale model should also be optimized to guide the “wet experiment”. Finally, protein engineering or enzyme‐directed evolution should be performed to enhance the affinity, specificity, and catalytic efficiency towards substrates. This will further improve the titre and selectivity of butyl butyrate, and reduce the downstream separation cost.

To reduce production cost, renewable feedstocks, such as lignocellulosic biomass (Cui et al., 2023; Feng et al., 2021; Lu et al., 2023) and cassava starch (Guo, Li, et al., 2024) have been used to produce renewable butyl butyrate. However, the titers were lower than those obtained with pure sugar. Therefore, more efforts should be made to increase butyl butyrate production from renewable feedstocks. Finally, to maximize the microbial production capacity, a continuous fermentation mode coupled with in‐situ extraction should be developed for industrial production of bio‐based butyl butyrate.

## AUTHOR CONTRIBUTIONS

**Xiaolong Guo:** Conceptualization; writing – original draft; writing – review and editing. **Yuqing Ding:** Writing – original draft; writing – review and editing. **Yufan Chen:** Writing – review and editing. **Hongxin Fu:** Conceptualization; writing – original draft; writing – review and editing; funding acquisition; supervision. **Jufang Wang:** Conceptualization; supervision; writing – original draft; writing – review and editing; project administration; funding acquisition.

## FUNDING INFORMATION

This work was supported by the National Natural Science Foundation of China (22178133), the Natural Science Foundation of Guangdong Province of China (2023A1515030100) and the Fundamental Research Funds for the Central Universities (2023ZYGXZR049).

## CONFLICT OF INTEREST STATEMENT

The authors declare no conflicts of interest.

## Data Availability

This article is an opinion, data sharing is not applicable to this article as no new data were created or analyzed in this study.
